# Prevalence and risk factors of sarcopenia in patients on maintenance hemodialysis: a retrospective cohort study

**DOI:** 10.1186/s12891-024-07546-3

**Published:** 2024-05-31

**Authors:** Qianyun Zhao, Yuyu Zhu, Xin Zhao, Rui Shi, Tingting Lu, Ran Yu, Deguang Wang

**Affiliations:** 1 Department of Nephrology, The Second Affiliated Hospital of Anhui Medical University, Hefei, Anhui Province China; 2 Lianhua Community Health Service Center of Hefei Economic and Technological Development Zone, Hefei, 230000 China

**Keywords:** Hemodialysis, Prevalence, Risk factor, Sarcopenia

## Abstract

**Background:**

This study aimed to explore the prevalence and related risk factors of sarcopenia in patients on maintenance hemodialysis (MHD).

**Methods:**

This cohort study enrolled 165 patients on MHD. The patients were divided into sarcopenia and non-sarcopenia groups based on the presence of sarcopenia or not. Sarcopenia was diagnosed according to the consensus of the Asian Sarcopenia Working Group that considers reduced muscle mass and decreased muscle strength (19). The muscle mass was measured using the multi-frequency bioelectrical impedance (Inbody260) and skeletal muscle index (SMI) was used: <7.0 kg/m^2^ (male); <5.7 kg/m^2^ (female) - with muscle mass reduction. The electronic grip dynamometer was used for measuring dominant handgrip strength (HGS) to reflect muscle strength. Male patients with HGS < 28 kg and female patients with HGS < 18 kg were considered with a decrease in muscle strength. The demographic characteristics, laboratory indexes, anthropometrical measurements, body compositions, and InBody score were compared between groups. The multivariate logistic regression was used to explore the risk factors for sarcopenia.

**Results:**

Of the 165 patients on MHD, 36 had sarcopenia, and the prevalence was 21.82%. Patients in the sarcopenia group had higher ages and lower body mass index, serum albumin level, circumference of waist, hip, and biceps, handgrip strength, total water content, protein inorganic salt concentrations, skeletal muscle mass, basal metabolic rate, obesity degree, SMI, and body fat content. The multivariate logistic regression showed that age, waist circumference, handgrip strength, and InBody score were influencing factors for sarcopenia in patients on hemodialysis.

**Conclusion:**

The prevalence of sarcopenia was high in patients on MHD. Higher age, lower waist circumference, lower handgrip strength, and lower InBody score were independent risk factors for sarcopenia in such patients.

## Background

Maintenance hemodialysis (MHD) is one of the most effective treatments for patients with end-stage renal diseases. It can effectively eliminate toxins in the system and increase internal environmental stability. However, the deterioration of renal function, metabolic disturbance, malnutrition, micro-inflammatory state, dialysis-related complications, and poor lifestyle, along with long-term hemodialysis, could accelerate the reduction in strength and mass of skeletal muscles and increase the risk of sarcopenia [1]. Sarcopenia, also known as age-related loss of skeletal muscle, is a degenerative disease associated with increasing age, which is mainly characterized by the reduction in skeletal muscle mass, strength, and function [2]. Muscle strength reduction, selective muscular structure change, and evident amyotrophy can occur with the deterioration of renal function in patients on MHD; the incidence of uremic sarcopenia is extremely high. Kim et al. [3] and Lamarca et al. [4] reported that the incidence of uremic sarcopenia was approximately 37.3–63.0%. The occurrence of sarcopenia could severely influence the outcomes of patients on MHD [5, 6]. It induced muscle strength reduction, decrease in muscle function, balance in capacity impairment, difficulties in walking and standing, high propensity for falls and disability, anxiety, and depression, consequently substantially decreasing the quality of life of patients on MHD [7–10]. The occurrence of sarcopenia could also increase the incidence of adverse events, such as cardiovascular events or even death [11], thereby significantly increasing the mortality in patients on MHD and medical expenditures [12].

Early effective interventions could prevent, delay, or even reverse sarcopenia [2]. Previous studies demonstrated that oral nutritional support and resistance exercises at least twice per week for patients on hemodialysis could enhance the nutrition state, muscle quality, and muscle strength, consequently improving the sarcopenia-related adverse outcomes [13–15]. However, there is a lack of awareness and concern about sarcopenia among medical staff and patients in most hemodialysis centers, therefore, the onset and progression of sarcopenia is not promptly diagnosed and treated. The incidence of sarcopenia in patients on MHD continues to increase every year, which can even induce cardiac or pulmonary failure, thus substantially endangering the safety of patients [16]. Therefore, medical workers should pay more attention to the detrimental effects of sarcopenia in patients on MHD, understand the prevalence and risk factors for sarcopenia in patients on MHD, and identify and provide interventions on the risk factors for sarcopenia at the earliest, which will ultimately effectively decrease the incidence and progression of sarcopenia in patients on MHD [17].

In this study, all patients who underwent regular hemodialysis were comprehensively screened to explore the prevalence of sarcopenia and investigate the risk factors. The findings of this study might provide evidence of preventing the occurrence of sarcopenia and improving the progression of sarcopenia in patients on MHD.

## Methods

### Study participants

The present study is a retrospective cohort study. Patients who underwent MHD in the blood purification center of The Second Affiliated Hospital of Anhui Medical University between July 2021 and July 2022 were included in this study. Maintenance hemodialysis is the principle of dispersion and convection to remove metabolic waste in blood, harmful substances and excessive water are a renal replacement therapy for patients with end-stage renal disease [18]. The study was approved by the Ethics Committee of the Second Hospital of Anhui Medical University (PJ-YX2020-006), and all patients signed an informed consent voluntarily.

The inclusion criteria were as follows: (1) patients ages ≥ 18 years; (2) patients who underwent regular hemodialysis for more than 3 months with the dialysis regimen of thrice per week and 4 h per dialysis; and (3) patients who underwent at least one anthropometrical measurement and comprehensive blood examination in the last 3 months.

**Fig. 1:**
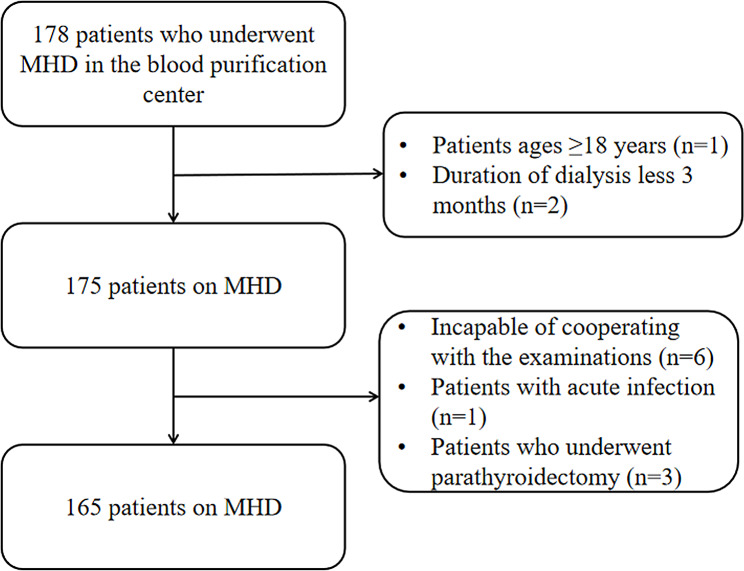
The exclusion criteria

The exclusion criteria were as follows: (1) patients with amputation of lower limbs, who could not stand up for bioelectrical impedance test; (2) patients with poor compliance or incapable of cooperating with the examinations; (3) patients with malignant tumors or cognitive impairment; (4) patients with acute infection or cardiovascular or cerebrovascular accidents in the last 6 months; and (5) patients who underwent parathyroidectomy (Fig. 1).

### Data collection

The demographic and clinical data of patients, such as age, sex, hemodialysis duration, and primary disease of MHD were collected by reviewing the medical records.

### Laboratory examinations

Fasting venous blood was collected from the patients before hemodialysis for the examinations of complete blood count, liver and renal functions, electrolytes, intact parathyroid hormone (iPTH), alkaline phosphatase (ALP), 10 indicators of immunity, β_2_-microglobulin, iron metabolism, blood glucose, and blood lipids. The venous blood after the hemodialysis was also collected. The renal function was assessed, and the urea clearance index (*Kt*/*V*) was calculated to assess dialysis adequacy.

### Anthropometrical measurements

After hemodialysis, four uniformly trained nurses with working experience of more than 10 years measured the height, dry weight, waist circumference, hip circumference, and handgrip strength. We measured biceps circumference and thickness of triceps using the tape measure and the body fat clip (Fresenius Kabi, Germany). The BMI and waist-to-hip ratio were calculated.

### Handgrip strength

The electronic grip dynamometer (EH101; Guangdong, China) was used for measuring dominant hand handgrip strength (HGS) to reflect muscle strength. The HGS on the contralateral side was measured in patients with internal fistula. The dominant HGS was measured in patients with long-term catheterization [1]. The patient took a sitting or standing position, with the arm on the non-fistula side resting flat on the table or a flat support, the palm facing upwards, and extending naturally [2]. Utilization of Grip Force: The tester placed the grip force device precisely in the center of the patient’s palm. The patient then firmly gripped the device, attempting to synchronize the efforts of both the thumb and the other four fingers, while sustaining the maximum grip force for a few seconds [3]. Reading and Recording: Once the patient had fully exerted their force, the testers ensured that the reading on the gripper was promptly and accurately captured. This process was repeated two to three times to calculate the average value.

### Muscle mass measurement

The body composition was measured using the multi-frequency bioelectrical impedance technique (body composition analyzer, Inbody260; Shanghai, China) within 20 min of hemodialysis in patients on MHD after the bladder was emptied. In brief, the patients removed all the metallic materials, took off the shoes and socks, and then stood on the machine. The body weight of the patients was measured. The patients placed the feet on the electrode plates and held the handles by hands with the thumbs on the electrode plates of handles, with angles between the arms and body. The age, sex, and height of the patients were recorded before the test. The patients remained still and relaxed during the test. Each test was completed in approximately 15 s. The impedance, body fat percentage, and muscle mass index were recorded at 20 and 100 Hz. All the tests were performed strictly following the protocols of the blood purification center. The temperature of the environment was kept constant at 22–26 °C to avoid the bias from thermal effects.

The InBody score is based on the difference between the measured values of muscle mass and fat mass and the standard values. The basic score is 80 points. If the muscle mass measurement value is lower than the standard value, points will be deducted accordingly. If it is higher than the standard value, points will be added. If the measured fat content is greater than or less than the standard value, points will be deducted. The passing score for InBody is 70 points for specialized individuals, 70–90 points are for the general normal population, and above 90 points are considered to indicate well-developed muscles.

### Diagnostic criteria of sarcopenia

Sarcopenia was diagnosed according to the 2019 consensus update issued by the Asian Working Group for Sarcopenia [19], and patients with muscle mass reduction and a decrease in muscle strength were diagnosed with sarcopenia. Male patients with a skeletal muscle index (SMI) < 7.0 kg/m^2^ and female patients with SMI < 5.7 kg/m^2^ were considered with muscle mass reduction. Male patients with HGS < 28 kg and female patients with HGS < 18 kg were considered with a decrease in muscle strength.

### Statistical analysis

The SPSS Statistics 23.0 software was used for the statistical analysis. Quantitative data with normal distribution were reported as means and standard deviations and compared using the independent-sample *t* test. Quantitative data without normal distribution were reported as median and interquartile range (IQR) and compared using the Wilcoxon rank-sum test. Qualitative data were reported as numbers and percentages and compared using the chi-square test followed by the Bonferroni adjustment to further explore the differences. The univariate binary logistic regression was used to explore the factors associated with the occurrence of sarcopenia in patients on MHD and screen the variables with statistical significance (*P* < 0.05). Collinearity diagnosis was performed for the variables with statistical significance, and the ones with severe multicollinearity [variance inflation factor (VIF) > 10] were excluded, while the other variables with statistical significance were included for multivariate logistic regression with the mode of “enter.” All tests were two sided, and *P* < 0.05 indicated a statistically significant difference.

## Results

### Comparison of general characteristics and laboratory findings

Of the 165 patients on MHD meeting the eligibility criteria, 36 (21.82%) were diagnosed with sarcopenia. Of patients on MHD ages ≥ 60 years, 24 had sarcopenia, and the prevalence was 14.55%. However, of patients ages < 60 years, 12 had sarcopenia, and the prevalence was 7.27%. Compared with the non-sarcopenia group, the age of patients in the sarcopenia group (64.6 ± 9.5) was significantly higher (*P* < 0.001). The albumin level in the sarcopenia group was 37.65 ± 2.83 g/L, which was significantly lower than that in the non-sarcopenia group (*P* < 0.001) (Table 1).

**Table 1:** Comparison of the general characteristics and laboratory findings between the sarcopenia and non-sarcopenia groups

General characteristics		Sarcopenia group (*n* = 36)	Non-sarcopenia group (*n* = 129)	t/Z	*P*
Age (year)		64.6 ± 9.5	54.9 ± 12.4	–5.072	0.001^***^
Male, *n* (%)		23 (63.89)	72 (55.81)	0.751	0.386
Hemodialysis duration (month)		89.6 ± 70.2	83.0 ± 55.2	–0.529	0.600
Primary disease *n* (%)	Chronic nephritis	8 (22.78)	35 (27.13)	1.615	0.656
	Diabetic nephropathy	7 (19.44)	17 (13.18)		
	Hypertensive nephropathy	12 (33.33)	51 (39.53)		
	Others	9 (25.00)	26 (20.16)		
Blood pressure before hemodialysis (mm Hg)	Systolic pressure	130.17 ± 18.00	130.99 ± 15.84	0.250	0.804
	Diastolic pressure	72.72 ± 9.42	76.17 ± 10.47	1.894	0.063
iPTH (pg/mL)		217.00 (56.73–611.25)	295.00 (160.00–491.50)	0.580	0.564
Albumin (g/L)		37.65 ± 2.83	39.61 ± 3.85	3.358	0.001^***^
Serum creatinine before hemodialysis (mmol/L)		21.42 (18.74–26.59)	22.87 (19.30–27.54)	–0.531	0.599
Blood calcium (mmol/L)		2.15 (2.05–2.29)	2.18 (2.05–2.28)	–0.875	0.387
Blood phosphorus (mmol/L)		1.82 ± 0.58	1.83 ± 0.54	0.170	0.866
ALP (U/L)		90.00 (79.00–21.00)	92.00 (71.00–27.00)	–0.471	0.640
Ferritin (g/L)		129.00 (34.50–204.00)	124.00 (61.05–226.50)	–0.792	0.428
*Kt*/*V*		1.80 (1.51–2.05)	1.62 (1.36–1.86)	–0.525	0.604
Transferrin (g/L)		2.00 (1.63–2.20)	2.00 (1.60–2.30)	1.022	0.309
β2-microglobulin (mg/L)	Before hemodialysis	30.70 (24.08–35.00)	30.20 (25.75–34.70)	1.051	0.295
	After hemodialysis	11.40 (10.20–13.70)	11.60 (10.20–14.00)	–0.940	0.353
Hemoglobin (g/L)		112.97 ± 18.53	111.72 ± 14.88	–0.373	0.710
CRP (mg/L)		1.70 (0.01–2.90)	2.40 (0.60–4.80)	–1.706	0.088

ALP, Alkaline phosphatase; CRP, C-reactive protein; *Kt*/*V* (URR), dialysis effectiveness index; PTH, parathyroid hormone. ^***^P < 0.001

### Comparison of bioelectrical impedance between the two groups

The BMI (20.93 ± 2.76 vs. 23.75 ± 3.76, *P* < 0.001), waist circumference (83.11 ± 10.32 vs. 91.10 ± 12.42, *P* < 0.001), hip circumference (90.28 ± 7.14 vs. 96.73 ± 11.27, *P* < 0.001), biceps circumference (24.18 ± 3.00 vs. 26.59 ± 4.99, *P* = 0.006), handgrip strength (7.15 ± 5.53 vs. 25.71 ± 8.56, *P* < 0.001), total water content (28.26 ± 4.37 vs. 33.15 ± 6.35, *P* < 0.001), protein concentration [7.35 (6.45–8.38) vs. 8.80 (7.55–9.95), *P* < 0.001], inorganic salt content (2.65 ± 0.35 vs. 3.02 ± 0.58, *P* < 0.001), body fat content (15.95 ± 6.10 vs. 18.96 ± 7.90, *P* = 0.017), skeletal muscle mass [20.59 (17.37–23.05) vs. 24.10 (20.60–28.0), *P* < 0.001], basal metabolic rate (1199.06 ± 126.43 vs. 1337.60 ± 177.79, *P* < 0.001), obesity degree (94.61 ± 11.98 vs. 108.54 ± 17.50, *P* < 0.001), InBody score (64.03 ± 6.42 vs. 67.04 ± 7.55, *P* = 0.025), and SMI (5.75 ± 0.79 vs. 6.83 ± 1.01, *P* < 0.001) were significantly lower in the sarcopenia group compared with that in the non-sarcopenia group (Table 2).

**Table 2:** Comparison of anthropometrical measurements and bioelectrical impedance between the sarcopenia and non-sarcopenia groups

Indicator	Sarcopenia group	Non-sarcopenia group	t/Z	*P*
BMI (kg/m^2^)	20.93 ± 2.76	23.75 ± 3.76	4.974	0.001^***^
Waist circumference (cm)	83.11 ± 10.32	91.10 ± 12.42	3.916	0.001^***^
Hip circumference (cm)	90.28 ± 7.14	96.73 ± 11.27	3.252	0.001^***^
Biceps circumference (cm)	24.18 ± 3.00	26.59 ± 4.99	2.764	0.006^**^
handgrip strength (cm)	7.15 ± 5.53	25.71 ± 8.56	5.678	0.001^***^
Thickness of triceps (cm)	10.75 (7.12–14.88)	13.00 (9.10–16.85)	–1.707	0.088
Waist-to-hip ratio	0.90 (0.86–0.98)	0.94 (0.90–0.98)	–1.365	0.172
Total water content (L)	28.26 ± 4.37	33.15 ± 6.35	4.344	0.001^***^
Protein (kg)	7.35 (6.45–8.38)	8.80 (7.55–9.95)	–4.531	0.001^***^
Inorganic salt (kg)	2.65 ± 0.35	3.02 ± 0.58	3.629	0.001^***^
Body fat content (kg)	15.95 ± 6.10	18.96 ± 7.90	2.447	0.017^*^
Skeletal muscle (kg)	20.59 (17.37,23.05)	24.10 (20.60,28.0)	-4.476	0.001^***^
Basal metabolic rate (kcal)	1199.06 ± 126.43	1337.60 ± 177.79	5.278	0.001^***^
Degree of obesity (%)	94.61 ± 11.98	108.54 ± 17.50	4.486	0.001^***^
InBody score (points)	64.03 ± 6.42	67.04 ± 7.55	2.307	0.025^*^
SMI (kg/m^2^)	5.75 ± 0.79	6.83 ± 1.01	6.819	0.001^***^

BMI, Body mass index; SMI, skeletal muscle index; ^*^P < 0.05; ^**^P < 0.01; ^***^P < 0.001

### Risk factors for sarcopenia

Binary logistic regression analysis was performed using the occurrence of sarcopenia as the dependent variable, while the aforementioned variables with *P* < 0.05, including age, serum albumin, BMI, waist circumference, hip circumference, biceps circumference, handgrip strength, total water content, protein concentration, inorganic salt content, skeletal muscle mass, basal metabolic rate, obesity degree, InBody score, SMI, and body fat content, were included as the independent variables. The findings showed that age was a risk factor for sarcopenia. Further, serum albumin level, BMI, waist circumference, hip circumference, biceps circumference, handgrip strength, total water content, protein concentration, inorganic salt content, skeletal muscle mass, basal metabolic rate, obesity degree, InBody score, SMI, and body fat content were protective factors for sarcopenia (*P* < 0.05) (Table 3).

**Table 3:** Univariate regression analysis of the factors associated with sarcopenia in patients on MHD

Indicator	Wals	OR	95% CI	*P*
Age	15.452	1.078	1.038–1.118	0.001^***^
Serum albumin	7.145	0.871	0.788–0.964	0.008^**^
BMI	14.612	0.791	0.702–0.892	0.001^***^
Waist circumference	10.893	0.944	0.913–0.977	0.001^***^
Hip circumference	9.320	0.940	0.904–0.978	0.002^**^
Biceps circumference	6.828	0.898	0.829–0.973	0.009^**^
handgrip strength	22.252	0.865	0.814–0.918	0.001^***^
Total water content	15.428	0.850	0.784–0.922	0.001^***^
Protein concentration	17.255	0.516	0.377–0.705	0.001^***^
Inorganic salt content	11.031	0.247	0.108–0.564	0.001^***^
Skeletal muscle mass	16.759	0.806	0.726–0.893	0.001^***^
Basal metabolic rate	15.370	0.994	0.992–0.997	0.001^***^
Obesity degree	16.198	0.946	0.921–0.972	0.001^***^
InBody score	4.234	0.945	0.895–0.997	0.04^*^
SMI	23.843	0.286	0.173–0.473	0.001^***^
Body fat content	4.304	0.947	0.899–0.997	0.038^*^

OR, Odds ratio; CI, confidence interval. The measurement units for the indicators listed in the first column are the same as in Table 2. ^*^P < 0.05; ^**^P < 0.01; ^***^P < 0.001

The occurrence of sarcopenia was included as the dependent variable, and age, serum albumin level, BMI, waist circumference, hip circumference, biceps circumference, handgrip strength, total water content, protein concentration, inorganic salt content, skeletal muscle mass, basal metabolic rate, obesity degree, InBody score, SMI, and body fat content were included for collinearity diagnosis. The result showed severe multicollinearity of BMI, total water content, inorganic salt, basal metabolic rate, obesity degree, SMI, and body fat content with other variables (VIF > 10), and thus these variables were excluded. Then, age, serum albumin level, waist circumference, hip circumference, biceps circumference, handgrip strength, protein concentration, skeletal muscle mass, and InBody score were included in the multivariate logistic regression analysis. The findings showed that age (*P* = 0.004, OR = 1.096, 95% CI = 1.030–1.165), waist circumference (*P* = 0.031, OR = 0.910, 95% CI = 0.836–0.991), handgrip strength (*P* < 0.001, OR = 0.825, 95% CI = 0.739–0.922), and InBody score (*P* = 0.004, OR = 0.855, 95% CI = 0.769–0.951) were independent influencing factors for sarcopenia occurrence in patients on MHD (*P* < 0.05) (Table 4).

**Table 4:** Multivariate regression analysis of the factors associated with sarcopenia in patients on MHD

	Wals	OR	95% CI	*P*
Age	8.484	1.096	1.030–1.165	0.004^**^
Albumin	0.845	0.926	0.785–1.091	0.358
Waist circumference	4.663	0.910	0.836–0.991	0.031^*^
Hip circumference	1.536	0.926	0.821–1.046	0.215
Biceps circumference	1.514	0.911	0.785–1.057	0.218
handgrip strength	11.644	0.825	0.739–0.922	0.001^***^
Protein concentration	0.239	0.964	0.830–1.118	0.625
Skeletal muscle	0.177	1.010	0.965–1.057	0.674
InBody score	8.387	0.855	0.769–0.951	0.004^**^

OR, Odds ratio; CI, confidence interval. The measurement units for the indicators listed in the first column are the same as in Table 2. ^*^P < 0.05; ^**^P < 0.01; ^***^P < 0.001

## Discussion

In this study, the 2019 consensus update issued by the Asian Working Group for Sarcopenia [19] was used to diagnose sarcopenia, which showed that the prevalence of sarcopenia in patients on MHD in our hospital was 21.82%. The prevalence of sarcopenia was 14.55% and 7.27% in patients on MHD ages ≥ 60 years and < 60 years, respectively. The risk of sarcopenia was higher in patients on MHD with higher ages. Moreover, chronic diseases and nutrition status were also closely associated with the occurrence of sarcopenia [19, 20]. The prevalence of sarcopenia was also relatively high in patients on MHD ages < 60 years. The findings of this study showed that age was a risk factor for sarcopenia occurrence in patients on MHD, whereas waist circumference, handgrip strength, and InBody score were protective factors for sarcopenia occurrence, indicating that patients on MHD with higher ages, lower waist circumference, lower handgrip strength, and lower InBody scores had a higher risk of sarcopenia.

The findings of this study showed that the risk of sarcopenia increased significantly with increasing age. The aging of muscles is a continuous process; the size and number of muscular fibers decrease progressively since the age of 25 years, and the muscle mass is reduced by approximately 30% at the age of 80 years [21]. The mass, strength, and synthetic capability of muscles decrease with the increase in age [22]. Further, various aging-related factors, such as changes in the levels of growth hormone and gonadal hormones, reduced protein intake, and an increase in the levels of inflammatory biomarkers, were associated with the occurrence and progression of sarcopenia [23]. The pathogeneses of sarcopenia are still unclear, which can be the result of the combined effects of multiple factors such as activation of inflammatory factors, reduction of mitochondrial functions, and body mass loss [24]. MHD could prolong the survival time of patients. However, various factors, including the loss of proteins during hemodialysis, increase in the levels of pro-inflammatory factors, metabolic acidosis, and reduced intake of proteins, could lead to the reduction of muscle strength, selective change in muscle structure, and evident amyotrophy with increasing age, continuous hemodialysis, and further worsening of renal functions in patients on MHD; thus, the risk of sarcopenia was extremely high in them [25]. A previous study showed that the prevalence of sarcopenia in Asian patients on hemodialysis (mean age 68 years) was 59.6% [8]. Therefore, this suggests that it is necessary to focus on the older MHD patients in clinic, which may help the prevention and early intervention of sarcopenia.

A new method, that is, the weight-adjusted waist index (WWI) reflecting the association between waist circumference and body weight, was suggested by experts from the Internal Medicine Department of the College of Medicine, Seoul National University [26]. The findings showed that WWI was positively correlated with total abdominal fat area, visceral fat area, and percentage of total tissue fat, but negatively correlated with appendicular skeletal muscle mass. The increase in WWI was associated with the increase in fat percentage and reduction in muscle mass and number [26]. The findings were in agreement with the results of this study that lower waist circumference was associated with a higher risk of sarcopenia.

handgrip strength is an important indicator for the screening of sarcopenia. The diagnostic criteria for sarcopenia included not only the reduction of muscle mass but also the decrease in muscle strength. The multivariate regression analysis in this study showed that handgrip strength, serving as the criterion for assessing muscle strength, was an independent influencing factor for sarcopenia, and patients with lower handgrip strength had a higher risk of sarcopenia.

The InBody score is assessed based on the differences in the means of muscle mass and fat content with the reference values. No previous studies investigated the direct association between InBody score and sarcopenia. This study showed that the InBody score was an independent influencing factor for sarcopenia, and lower InBody scores indicated a higher possibility of sarcopenia in patients on MHD. We hypothesized, based on the scoring criteria of InBody scores, that muscle mass was positively associated with the InBody score, and patients with lower muscle mass had lower InBody scores and a higher risk of sarcopenia. The body fat content in the reference range was considered optimal, while the content higher or lower than the reference range could reduce the InBody score, thus increasing the risk of sarcopenia. Therefore, patients on MHD should be encouraged to perform moderate exercises and increase muscle mass. Moreover, a reasonable diet is also recommended. Further, patients should avoid either eating and drinking too much or over-restriction of diet to avoid too high or low fat content, which can lead to sarcopenia.

Previous studies showed that the incidence of sarcopenia was significantly higher in patients with diabetes [27]. The percentage of patients with diabetes was not significantly different between the two groups, which could be associated with the sample size and different diagnostic criteria used in the studies.

This study was a single-center cross-sectional study with a relatively small sample size. Various factors, such as diets, exercise, and social–psychological factors, could influence muscle mass and strength. However, this study only collected and analyzed the representative influencing factors, while additional relevant data were still lacking. The characteristic of sarcopenia occurrence in patients on hemodialysis need to be more extensively investigated through further clinical or fundamental studies with more rigorous study designs.

Nevertheless, it is worthy to point to several limitations of our study. On the one hand, the present study is a single-center retrospective study and was likely affected by selection bias. On the other hand, we only collected the baseline characteristics of the patients. Therefore, the relation between the dynamic changes in the sarcopenia and maintenance hemodialysis could not be analyzed.

## Conclusions

In summary, sarcopenia is relatively common in patients on MHD, while patients on MHD with sarcopenia have higher risks of fall and fracture; thus, the quality of life is reduced while the mortality rate is increased. The understanding of researchers globally on the occurrence of sarcopenia in patients on hemodialysis has increased in recent years, but the understanding of nursing workers and patients regarding sarcopenia is still insufficient. The nutritional assessment of patients on hemodialysis should be performed regularly to identify the risk factors for sarcopenia and thus it may help perform interventional measurements to prevent the occurrence or delay the progression of sarcopenia. This study aimed to investigate the prevalence and risk factors of sarcopenia in patients on MHD and provide evidence for the early diagnosis and prevention of sarcopenia in clinical practice.

## Data Availability

All data generated or analysed during this study are included in this published article.
